# Understanding racial disparities in severe maternal morbidity using Bayesian network analysis

**DOI:** 10.1371/journal.pone.0259258

**Published:** 2021-10-27

**Authors:** Mandana Rezaeiahari, Clare C. Brown, Mir M. Ali, Jyotishka Datta, J. Mick Tilford

**Affiliations:** 1 Department of Health Policy and Management, University of Arkansas for Medical Sciences, Little Rock, Arkansas, United States of America; 2 University of Arkansas for Medical Sciences, Institute for Digital Health & Innovation, Little Rock, Arkansas, United States of America; 3 Department of Statistics, Virginia Polytechnic Institute and State University, Blacksburg, Virginia, United States of America; Montclair State University, UNITED STATES

## Abstract

Previous studies have evaluated the marginal effect of various factors on the risk of severe maternal morbidity (SMM) using regression approaches. We add to this literature by utilizing a Bayesian network (BN) approach to understand the joint effects of clinical, demographic, and area-level factors. We conducted a retrospective observational study using linked birth certificate and insurance claims data from the Arkansas All-Payer Claims Database (APCD), for the years 2013 through 2017. We used various learning algorithms and measures of arc strength to choose the most robust network structure. We then performed various conditional probabilistic queries using Monte Carlo simulation to understand disparities in SMM. We found that anemia and hypertensive disorder of pregnancy may be important clinical comorbidities to target in order to reduce SMM overall as well as racial disparities in SMM.

## Introduction

Severe maternal morbidity (SMM) is an unexpected outcome of labor and delivery that results in short-term or long-term adverse health outcomes [1]. It is defined by the Centers for Disease Control and Prevention (CDC) to include 21 different indicators [2]. SMM affects approximately 50,000 women annually with a steadily increasing rate in recent years [3]. Additionally, SMM is an important predictor of maternal death [4].

Significant racial disparities exist in maternal mortality and SMM. SMM disproportionately affects women of minority race or ethnicity, especially non-Hispanic Black women, who have over twice the rates of SMM compared to non-Hispanic White women [5]. Black women are 3 to 4 times more likely to die from pregnancy-related causes compared to Non-Hispanic White women [6].

Disparities are defined by the American College of Obstetricians and Gynecologists as “differences that result in a particular type of health difference that is closely linked with economic, social, or environmental disadvantage” [7]. Social determinants of health play an important role in disparities in maternal morbidity and mortality, including individual factors (e.g., socioeconomic status, race and ethnicity, gender), community and neighborhood factors (e.g., social networks and built environment, housing), provider factors (e.g., knowledge, implicit bias, communication), and system factors (e.g., availability of and access to high quality care, structural racism, social and political policies) [8]. A wide range of factors have been shown to increase the likelihood of SMM including: increased maternal age, pre-pregnancy obesity, pre-existing chronic medical conditions and cesarean delivery [9]. Evaluating the complex relationship between various social determinants of health, maternal clinical characteristics and risk factors could help identify the potential drivers of racial disparities in rates of SMM.

The most widely used analytical method to study associations between risk factors and SMM is based on multiple regression models [10–12]. These methods estimate the association of each input variable with the outcome variable while holding all other input variables constant. The drawback of this approach is that it prevents the discovery of nuanced and complex relationships between the various input variables. Bayesian networks (BNs) have emerged as a popular statistical tool for building graphical models that represent stochastic dependence among random variables. The relationships between predictors are often unknown and must be learned from the data [13]. Previous research has used BNs to understand factors related to various health outcomes, and showed that BNs may be superior to classical (frequentist) analyses that use regression models [14].

A BN describes the joint probability distributions of a network of factors using a Directed Acyclic Graph (DAG), where “directed acyclic” means the directional relationships between factors cannot be cyclical in nature [15]. Factors that affect the likelihood of an outcome are represented by nodes, and the relationships among factors are represented using directed arcs. If the state of one factor (“A”) influences the state of the other factor (“B”), a directed arc is drawn between the two nodes from “A” to “B” [16]. The parameters and dependencies in the network can be learned from the data or provided by expert knowledge [17,18]. Additionally, BNs can be used to query any given node in the network, which allows for estimation of the probability distribution of the outcome given a specific value on that node (e.g., the probability of maternal morbidity given the mother lives in a rural area).

BNs have been successfully used as hypothesis-generating tools by elucidating the associations among variables using DAGs. Noyes et al. (2018) used BN to uncover the associations between the vaginal microbiome and risk of bacterial vaginosis [19]. The result of their study found newly documented associations between birth control usage, menstrual hygiene practices, and specific microbiome members. Their study uses BN as an exploratory data analysis tool rather than causal inference tool [19]. BNs have also been widely used in medical decision making due to the ability of BNs to encode uncertain domain knowledge in a natural manner [20]. Medical informatics has been driving the development of BNs due to the ability of BNs to intuitively encapsulate the causal links between diagnostic and prognostic factors within large, complex medical datasets [21,22]. In medicine, causal BNs have been used for clinical decision making using diagnostic or prognostic disease models [23–25]. The underlying causal models in diagnostic and prognostic causal models are constructed from the domain literature, expert knowledge, and statistical data [23]. Furthermore, studies have found that BNs that learn from relationships in the data (“unsupervised” or “semi-supervised” models) have comparable performance to models based on clinician-knowledge alone (“supervised”) [26]. One caveat of the causal inference models is the risk of expert knowledge misleading statistical adjustment which may lead to the occurrence of “paradoxes.” An important example is the “birth weight paradox” that occurs when stratifying on the birth weight creates spurious association between the exposure (maternal smoking) and outcome (infant mortality). In other words, the paradox occurs when an intermediate variable (birth weight) is on the causal pathway of the exposure (maternal smoking) and the outcome (infant mortality) and shares common causes with the outcome (infant mortality) [27]. Therefore, it is essential to clarify the causal question and the assumptions regarding the causal structure for any analytical approach [27]. It is worth pointing out that association mining methods such as BN has a potential risk of runing into paradox in presence of unobserved confounding variables that could reverse the direction of association. However, the BN approach proposed in this study is inductive and does not use DAG to seek any causal interpretations.

Additionally, unlike structure equation modeling (SEM) that quantifies the slope of the effect based on a predetermined structure, the proposed BN in this study deploys a data-driven approach to understand the structure of the associations among the variables [28]. In other words, the BN structure identifies which variables have a direct effect on developing SMM and which variables, on the other hand, have their effect on SMM mediated by other input variables. In this study, our goal is to assess the total effect of race on the occurrence of SMM based on the learned BN structure. Previously published studies [29] provide further information related to mediators on the causal pathways. Bayesian learning of BN in this study is an approach to automatically building BNs from data then revising the BN structure (i.e., removing the nodes and/or arcs) based on prior knowledge (semi-supervised).

For this study, birth certificate data was linked to the Arkansas All-Payer Claims Database (APCD), which gave us a rich source of information on maternal demographic and clinical characteristics, including comorbidities and SMM.

## Materials and methods

A major motivation for using BN in this paper was to develop a statistically rigorous and intuitively understandable model. The first step was to identify factors associated with SMM, and the second was to use BN to query various nodes in the network to understand disparities associated with SMM. In order to characterize the properties of BN-derived information, the first task was to choose the most robust network structure. This study was deemed non-human subjects research by the first author’s institution (#229073), and requirement of written or verbal informed consent was waived.

### Data

The primary data source for this study was the Arkansas APCD, years 2013–2017. The APCD, administered by the Arkansas Center for Health Improvement, contains data from multiple sources, including Medicaid and commercial insurance claims and Vital Statistics (i.e., Birth Certificate) data. The APCD includes membership/enrollment information and insurance claims for beneficiaries of state and federal health plans, the individual market, small and large employers, and a portion of self-insured employers [30]. Our study excluded claims from Medicare and from workers’ compensation, and included women with private/commercial coverage, Medicaid coverage, or coverage in a qualified health plan through the Arkansas Health Insurance Marketplace. This study was deemed non-human subjects research by the first author’s institution (#229073).

Females between the age of 12 to 55 with at least one birth between the years 2014 and 2017 were included. Although our data included years 2013 through 2017, births between April 1^st^, 2014 to November 19^th^, 2017 were included to ensure each woman had at least a 15-months look-back period (6 months prior to pregnancy and 9 months pregnancy period) as well as a follow-up period (42 days postpartum). As a result, 77,172 births were identified between April 1^st^, 2014 and November 19^th^, 2017.

For each birth, the comorbidities within the 15-months look-back period were identified. The comorbidities in our study included pre-existing conditions (prior to pregnancy or acquired during pregnancy) as described by Reid et al. [31] and Mhyre et al. [32]. Additional conditions previously used in studies on SMM were also included. Comorbidities were defined based on ICD-9 and ICD-10 diagnosis codes (see S1 Table for full list of codes).

In addition to clinical information from the claim files, several useful data elements were derived from the birth certificate file for each of the included births in this study. Area level information was obtained from the Area Health Resources Files based on the mother’s county of residence [33]. Table 1 also indicates the source of each covariate variable. All continuous variables were converted into discrete variables for the analysis. The four age categories were ages 15 to 19, 20 to 34, 35 to 39, and age 40 or older. County level factors, including percent of African-American, percent owner occupied houses, percent urban population, percent poverty, and number of OBGYN per 1000, were categorized based on quartiles.

**Table 1:** Variables and data resources in the study.

Birth certificate	Area Health Resource File	Medical Claims				
Maternal and birth-related variables	County-level variables	Conditions				
*Age* Less than 20 years 20 ≤ age < 35 35≤ age<40 age ≥ 40	*Primary care coverage* Partial shortage area No shortage area Whole shortage area	Multiple gestation	Chronic renal disease	Excessive weight gain in pregnancy	Malignancy	Spine abnormalities
*Race* White-non-Hispanic Black-non-Hispanic Hispanic Other	*Percent African-American quartile*	Insufficient prenatal care	Renal disease complicating pregnancy	Infections of genitourinary tract in pregnancy	Mental health disorders	Substance use Alcohol
*Education* At least some college Less than high school High school graduate Unknown education	*Percent owner occupied houses quartile*	Prior cesarean	Chronic respiratory disease	Habitual aborter currently pregnant	Mental disorders complicating pregnancy	Substance use Cannabis
*Birth Year* 2014 2015 2016 2017	*Percent urban population quartile*	Anemia	Coagulation defects complicating pregnancy	Hemorrhage in early pregnancy	Obesity	Substance use Cocaine
*Insurance* Private insurance Medicaid Self-pay Other	*Percent poverty quartile*	Asymptomatic bacteriuria in pregnancy	Congenital heart disease	Hepatitis C	Pregnancy-related Obesity	Substance use Opiates
*Marital status* Married Not married/Unknown	*Number of OBGYN per 1000 quartile*	Bariatric surgery status	Congenital cardiovascular disorders complicating pregnancy	Deep vein thrombosis	Other cardiovascular diseases complicating pregnancy	Substance use Other
*WIC program participation* Yes No Unknown		Bone and joint disorders	Diabetes mellitus complicating pregnancy	Hypercoagulable state	Peripheral neuritis in pregnancy	Substance use Unspecified
*Parity* 0 1 2 or more Unknown		Cervical shortening	Diabetes mellitus	Hypertensive disorder of pregnancy	Placenta previa	Systemic lupus erythematosus
		Chronic heart disease	Drug dependence complicating pregnancy	Infectious and parasitic conditions complicating pregnancy	Preexisting hypertension	Tobacco use disorder complicating pregnancy
		Chronic liver disease	Epilepsy complicating pregnancy	Liver and biliary tract disorders in pregnancy	Sickle cell disease	

In our study, SMM conditions were defined as the 21 indicators outlined by the CDC [2] and was measured during the period starting from the date of delivery through 42 days postpartum.

### Bayesian network

A BN is a graphical model of associations among a set of random variables that consists of two components:

1. a network structure depicted as a DAG. Within a DAG, each node represents a variable and the arc between two nodes represent the stochastic dependency between those two variables [34].
2. a set of conditional probability distributions for each variable within a DAG according to the stochastic dependencies represented by the edges (connections between two nodes). These conditional distributions are specified by the network parameters [35].

If there is an arc (directed edge) from node X_1_ to node X_2_ in a DAG, then X_1_ is said to be a parent of X_2_; likewise, X_2_ is called the child of X_1_. An important feature of BNs is the concept of Markov conditions which states that each variable represented by a node is conditionally independent of its predecessors given the values of its parents. Based on the Markov condition, the global joint probability of the entire BN can be represented by the product of conditional probabilities using the graphical structure and the chain rule of probability [34]:

$$p\left(x\text{|}\theta \right)=\prod_{i=1}^{n}p(x_{i}|pa\left(x_{i}\right),\theta_{i})$$ (1)

where *X* = {*x*_1_, *x*_2_,…,*x*_*n*_} and *θ* = {*θ*_1_, *θ*_2_,…, *θ*_*n*_} represent the variables (nodes) and the parameters in the BN, respectively. Each *θ*_*i*_ is the set of parameters necessary to specify the probability distribution of each variable (x_i_) given its parents (pa(x_i_)) [34].

Given a dataset D = {*D*_1_,…,*D*_*N*_}, where each data point D_i_ is a vector of values for variables X, structure learning is the task of finding a network structure that best fits D [36]. If D is discrete and complete then *θ* is maximized using frequency counts from the data [37]. The process of learning the DAG of a BN is a complex task, and the number of DAGs grows super-exponentially as the number of nodes grow. The process of learning in BN consists of two main steps: (1) learning the structure of the DAG and (2) parameter learning. Parameter learning is related to learning the local distributions implied by the structure of DAG learned in step 1 [38]. Several algorithms have been presented in the literature for structure learning based on the data which can be categorized in to three types: constraint-based, score-based, and hybrid techniques [38].

Constraint-based algorithms are based on the Inductive Causation (IC) algorithm developed by Pearl (1991) which uses conditional independence tests to learn the DAG [39]. The first step in the IC algorithm is to identify which variables are connected regardless of the direction of the relationship between the two variables. This step starts with a saturated model (i.e., a complete graph with all variables), and then the graph is pruned based on statistical tests for conditional dependence. The second step involves identifying the v-structures among all non-adjacent nodes A and B with a common neighbor C. A v-structure is the only structure in which non-adjacent nodes are dependent given a common neighbor in C. At the end of this step, the skeleton and v-structure of the BN is known. The last step, step 3, is related to orienting the arcs. Due to the computational costs associated with the IC algorithm, several improved and efficient algorithms have been developed [38]. Some of these improved algorithms include Grow-Shrink (GS) [40], Incremental Association (IAMB) [41], Fast Incremental Association (Fast-IAMB) [42] and Interleaved Incremental Association (Inter-IAMB) [41]. All of these algorithms first learn the Markov blanket of each node which simplifies the identification of neighbors and reduces the computational complexity of the learning algorithm.

The second structured learning approach, the score-based technique, applies the concept of optimization to the problem of structure learning where each candidate network structure is given a network score reflecting its goodness of fit, which is then maximized within the process of optimization [38]. Hill-climbing (HC), simulated annealing, genetic algorithm, tabu search (TS) are some examples within this class of algorithms [43].

Finally, hybrid algorithms combine constraint-based algorithms and score-based algorithms to compensate the respective weaknesses and produce a reliable network [38]. Max-Min hill-climbing (MMHC) [44], restricted maximization (rmax2) [45], and hybrid H2PC [46] are examples of hybrid algorithms.

**Fig 1:**
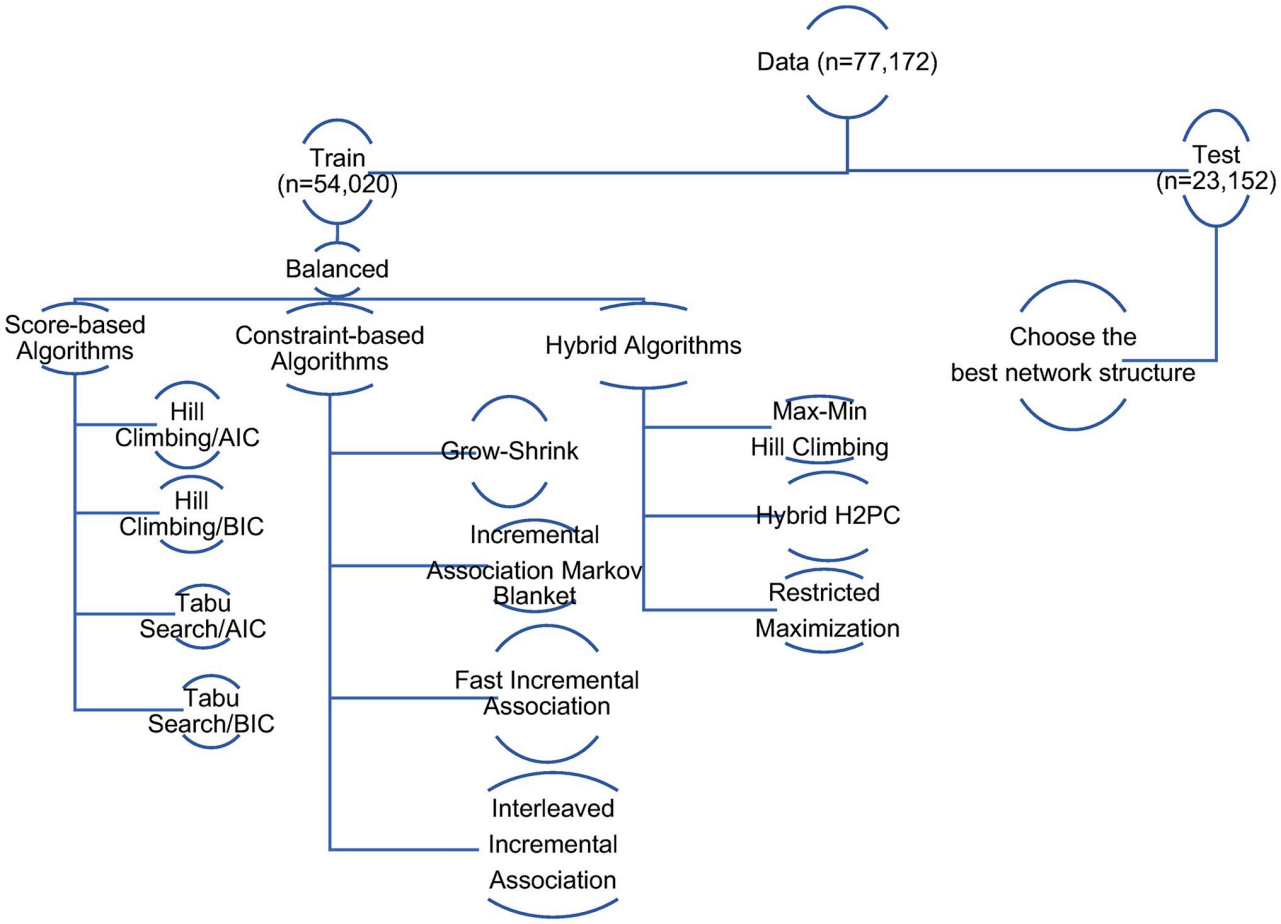
Procedure of structure learning in this study.

In this study, in order to learn the structure of the BN, a ‘validation set’ approach was used, i.e., data was randomly divided into training (70%) and test (30%) sets. The training set was then used to learn the BN structure, and the test set was used to evaluate the predictive performance of the learned structure. Throughout the learning step, we adopted a layering strategy as implemented in other studies to force the direction of some probabilistic relations [47,48]. In layering strategy, nodes are allowed to have relationship within the layer or with the variables in the lower layer but cannot have parents in the higher levels [47]. For example, mother’s race is allowed to be linked to diabetes mellitus complicating pregnancy, but the reverse is not allowed. Fig 1 summarizes the list of learning algorithms used in this study.

### Handling imbalanced data

A data set is called class-imbalanced if the number of observations in each category of the target (outcome) variable are significantly different. The dataset in this study is imbalanced as only 2% of the study population experienced SMM [49]. Such an extreme class imbalance in the binary response variable could potentially skew the inference and parameter estimates if not properly accounted for. To handle the extreme class imbalance, structure learning was performed using both balanced data and original data. Sampling is a widely used technique to mitigate imbalanced data where the distribution of observations is altered so that minority class is more adequately represented in the data [50]. Common sampling approaches to mitigate imbalance include random oversampling (with replacement) of the lesser prevalent class and random under sampling (without replacement) of the prevalent class [51].

In order to handle the class imbalance problem, random under sampling was employed on the training set. Random under sampling is a simple yet effective method that eliminates some instances of the majority class to create a nearly equal instances in both the minority and majority class [52]. The sample size of the unbalanced data (train and test sets) and the balanced dataset is given in Table 2.

**Table 2:** Number of SMMs in different datasets.

	SMM	No SMM
**Original**	1,755	75,417
**Train**	1,193	52,827
**Test**	562	22,590
**Under sampled data**	1,193	1,193

### Selection of robust Bayesian network

**Fig 2:**
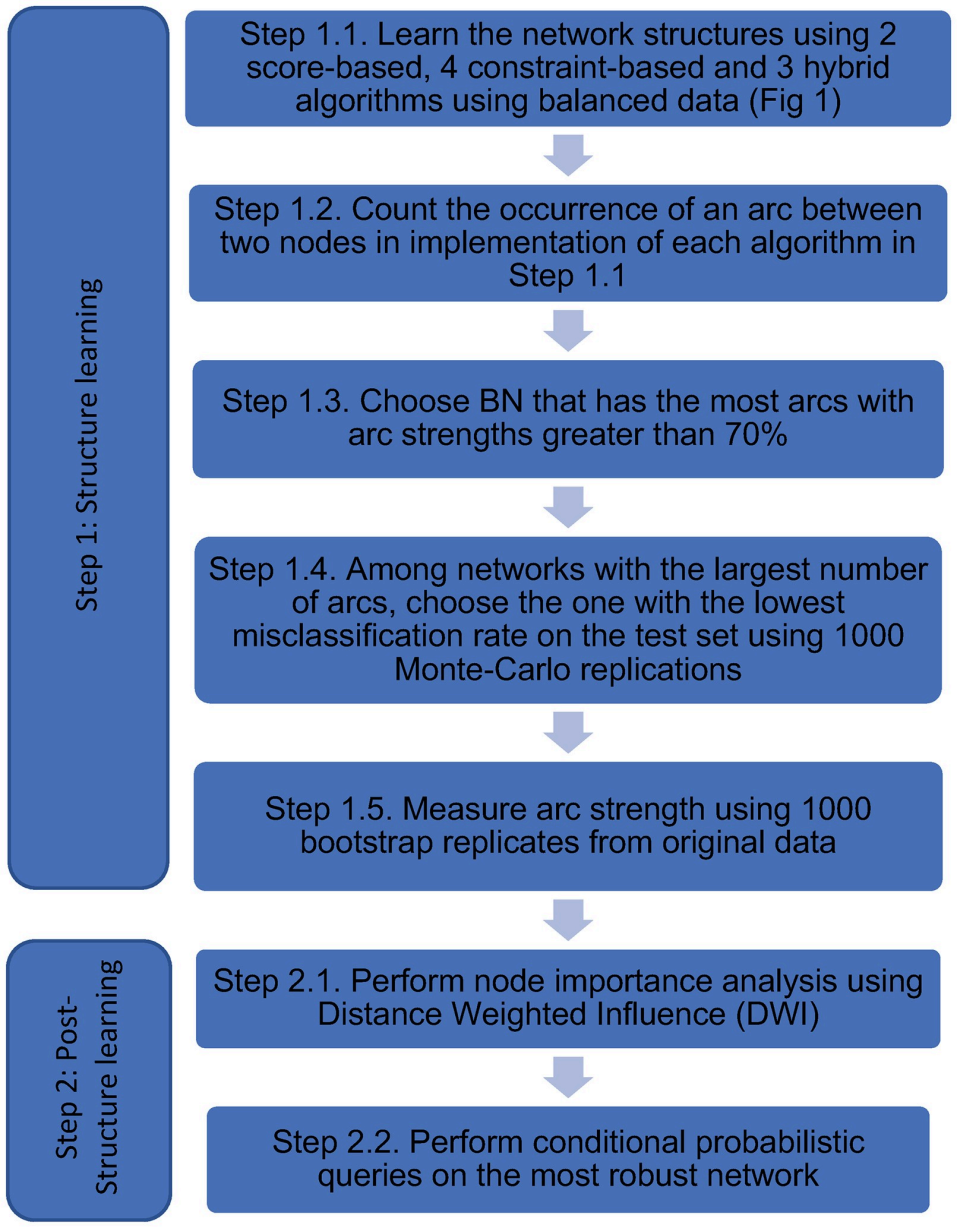
Procedure employed in this study.

There are limited techniques available in the literature for assessing the statistical robustness of network structures learned from data [53]. One logical approach used to study structural learning algorithms is to measure differences by using reference datasets. However, this approach fails when studying real-world datasets due to unknown underlying probability distributions [54]. One systematic approach to identify significant features in the network is based on using bootstrap resampling and model averaging [55]. In this approach the proportion of arc presence is used to derive the importance of an arc. In this study, the approach outlined by Cugnata et al. [54] is used as the basis to design a robust network. Fig 2 outlines the summary of the approach.

In the first step, the data is analyzed with eleven different learning structures (Fig 1) using the “bnlearn” package in R 1.2.5042 [56]. Learning algorithms included two score-based algorithms (HC with score functions AIC and BIC and tabu search with score functions AIC and BIC), four constraint-based algorithms (GS, IAMB, Fast-IAMB, Inter-IAMB), and three hybrid algorithms (MMHC, rmax2, hybrid H2PC).

### Node importance analysis

In step 2, we used a metric called Distance Weighted Influence (DWI) to rank the influence of query nodes on the SMM based on the network structure [57]. DWI is an influence of X on Y, DWI(X,Y;w) and is defined as:

$$DWI\left(X,Y;w\right)=\sum_{s\in S\left(X,Y\right)}w^{\left|s\right|}$$ (2)

where |*s*| is the length of the simple path s and 0 ≤ *w* ≤ 1. In Eq 2, the sum of coefficients is the sum of unblocked paths between X and Y, and the smallest power is the smallest length of an unblocked path between X and Y. DWI depends on the number of paths, and it decreases as the length of the paths increase [57].

## Results

Table 3 shows the occurrence of an arc between any two nodes that occurred more than 70% of the times in the implementation of each algorithm. In Table 3, a value of 1 indicates that there existed a directed arc between the two nodes while a value of 0.5 indicates that two nodes are linked with an undirected arc. The last column shows the sum of the arc strengths across all the eleven structure learning algorithms, with higher numbers suggesting a more common relationship between the two variables.

**Table 3:** Arc strengths obtained using different structure learning algorithms.

Arcs	HC-BIC	HC-AIC	TS-BIC	TS-AIC	GS	IAMB	Fast-IAMB	Inter-IAMB	MMHC	hybrid H2PC	rmax2	arcs occurrence
Hypertensive disorder of pregnancy →SMM	1	1	1	1	1	1	1	1	1	1	1	11
Parity→ Prior_CSec	1	1	1	1	1	1	1	1	1	1	1	11
Mental disorders complicating pregnancy → Infectious and parasitic conditions complicating pregnancy	1	1	1	1	0.5	1	1	1	1	1	1	10.5
mother_race→ married	1	1	1	1	1	1	0.5	1	1	1	1	10.5
Obesity → Pregnancy-related Obesity	1	1	1	1	0.5	1	1	1	1	1	1	10.5
Anemia →SMM	1	1	1	1	0	1	1	1	1	1	1	10
Other cardiovascular diseases complicating pregnancy →Chronic heart disease	1	1	1	1	1	1	0	1	1	1	1	10
Preexisting hypertension → Mental health disorders	1	1	1	1	0	1	1	1	1	1	1	10
Prior cesarean →SMM	1	1	1	1	0	1	1	1	1	1	1	10
Substance use Cannabis → Mental disorders complicating pregnancy	1	1	1	1	0	1	1	1	1	1	1	10
Substance use Opiates →Substance use Other	1	1	1	1	0.5	1	0.5	1	1	1	1	10
Percent African American →Percent Urban Population	1	1	1	1	0	1	0.5	1	1	1	1	9.5
Chronic renal disease → Renal disease complicating pregnancy	1	1	1	1	0.5	0.5	0.5	0.5	1	1	1	9
Infectious and parasitic conditions complicating pregnancy → Infections of genitourinary tract in pregnancy	1	1	1	1	0	1	1	1	1	1	0	9
Percent Poverty →Percent Urban Population	1	1	1	1	0	1	0	1	1	1	1	9
Substance Use Opiates →Substance use Unspecified	1	1	1	1	0	1	0	1	1	1	1	9
Coagulation defects complicating pregnancy → Hypercoagulable state	1	1	1	1	0.5	0.5	0	0.5	1	1	1	8.5
Hypertensive disorder of pregnancy → Excessive weight gain in pregnancy	1	1	1	1	0	0.5	0.5	0.5	1	1	1	8.5
Mental health disorders → Mental disorders complicating pregnancy	1	1	1	1	0.5	0	1	0	1	1	1	8.5
Anemia →Sickle cell	1	1	1	1	0	0.5	0	0.5	1	1	1	8
Diabetes mellitus complicating pregnancy → Diabetes mellitus	1	1	1	1	0	1	1	1	0	0	1	8
education →payer	1	1	1	1	0	1	0	1	1	1	1	8
Preexisting hypertension→ Infections of genitourinary tract in pregnancy	1	1	0	1	0	1	0	1	1	1	0	8
Infections of genitourinary tract in pregnancy → Asymptomatic bacteriuria in pregnancy	1	1	1	1	0.5	0	1	0	1	1	0	7.5
Mental health disorders →Chronic respiratory disease	1	1	1	1	0.5	0	0	0	1	1	1	7.5
Percent African American →Percent Owner Occupied House	1	1	1	1	0	0	0.5	0	1	1	1	7.5
Percent Owner Occupied House→ OBGYN per 1000	1	1	1	1	0	0	0.5	0	1	1	1	7.5
Percent Urban Population→OBGYN per 1000	1	1	1	1	0	0	0.5	0	1	1	1	7.5
Percent Urban Pop →Percent Owner Occupied House	1	1	1	1	0	0	0.5	0	1	1	1	7.5
Total Arcs	29	29	28	29	8	20	16.5	20	28	28	26	

**Fig 3:**
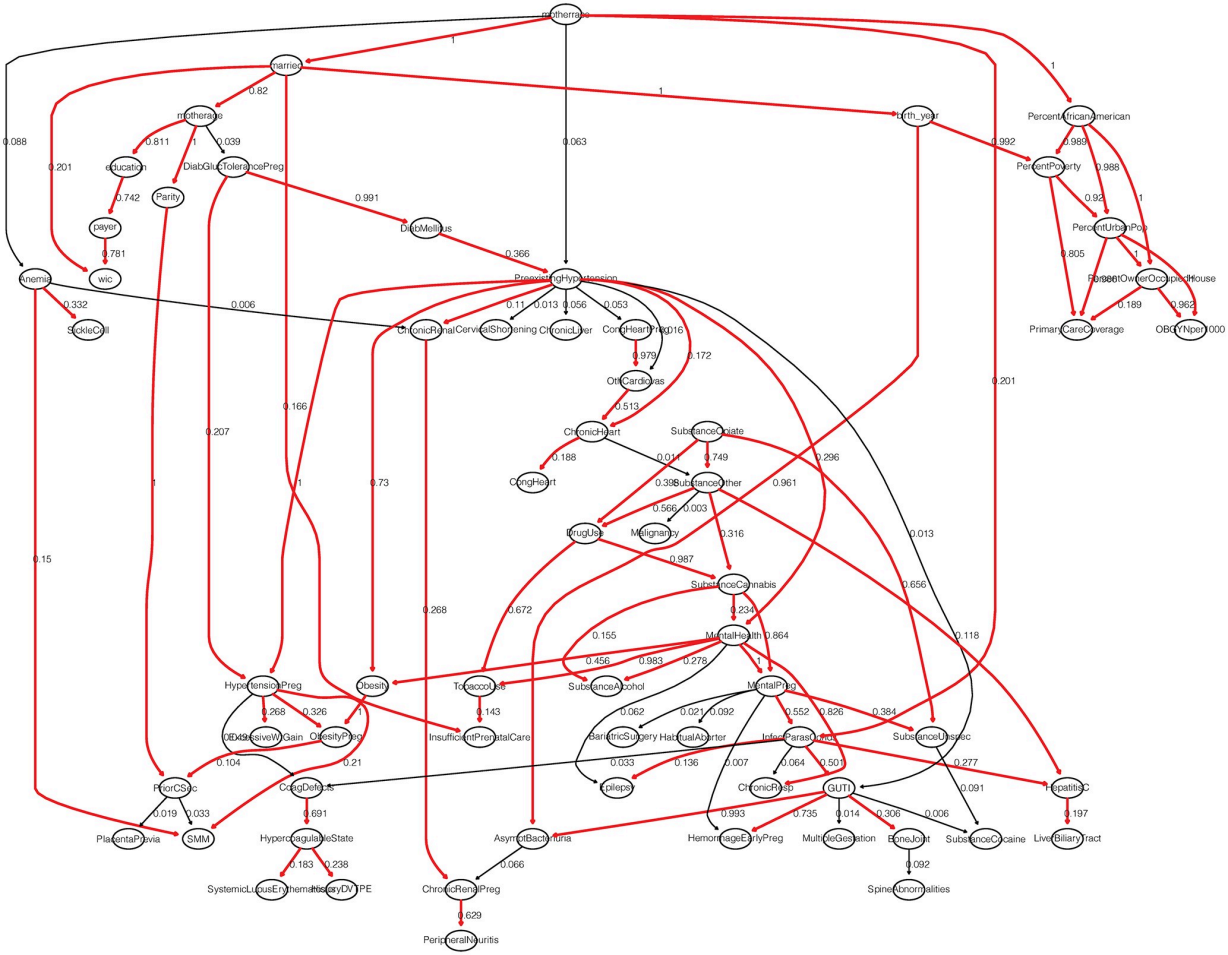
BN learned based on HC-BIC with proportion of occurrence of each arc in the bootstrap replicates.

We chose the structure learning algorithms with the greatest number of arcs and arc strengths greater than 70% across all structure learning algorithms. Therefore, the first three structure learning algorithms (HC-BIC, HC-AIC, TS-AIC) were selected. Since the choice of the most robust network does not guarantee that the network predicts efficiently [54], we trained each of the four algorithms based on 1000 Monte-Carlo replications in the training set and calculated the misclassifications rates using the test set of the original data. HC-BIC, HC-AIC, and TS-AIC achieved misclassification rates of 25%, 26%, and 27% using the test set. We then generated 1000 samples with 1000 observations from the original data and calculated the arc strength. Arc strength equals the proportions of occurrence of each arc for each bootstrapped sample using the algorithm with lowest misclassification, HC-BIC. Fig 3 shows the BN learned using HC-BIC and the arc strengths based on the average arc strength in 1000 bootstrapped samples. Of note is that the BN in Fig 3 is not a causal BN due to the possibility of unobserved variables.

**Fig 4:**
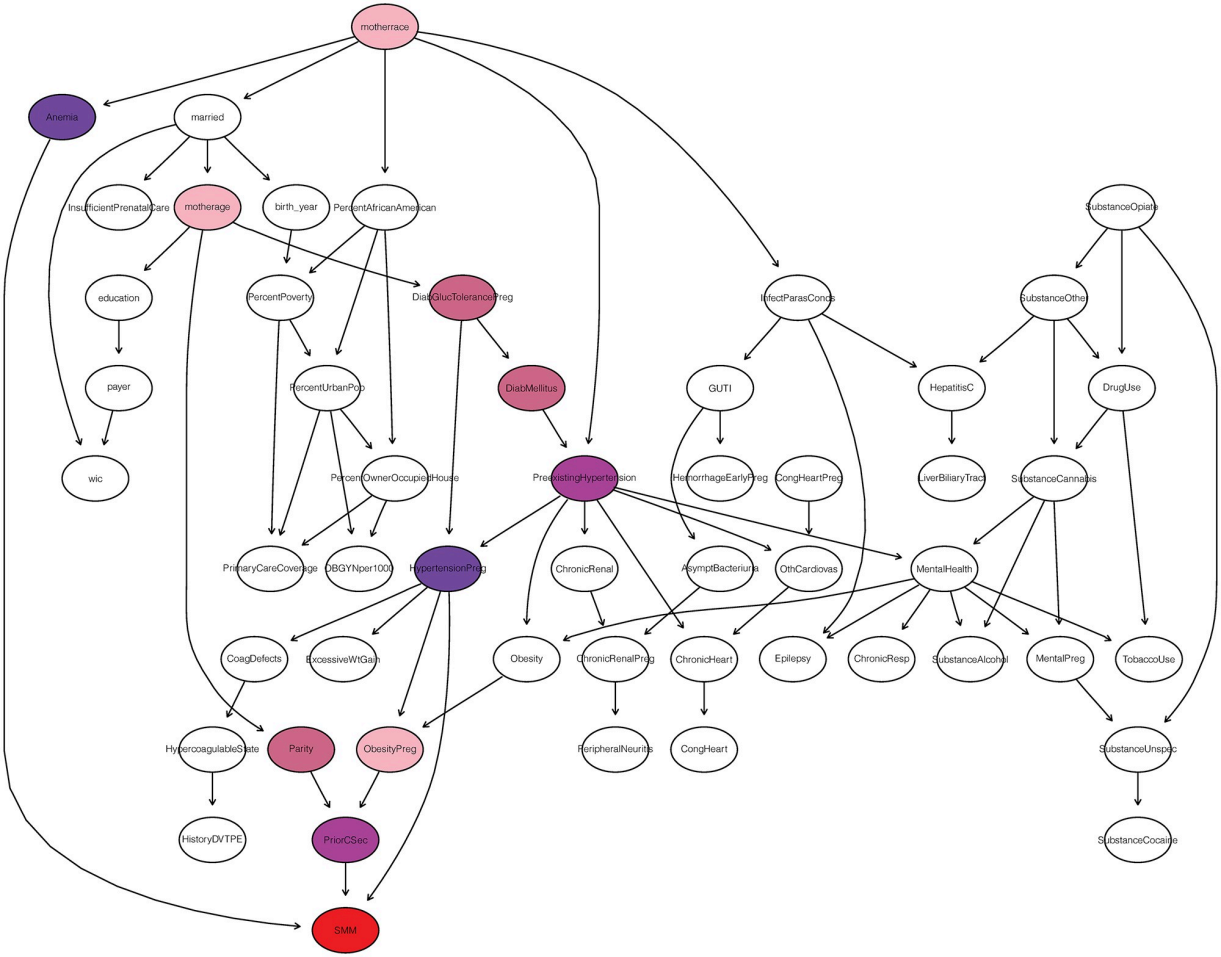
Heatmap of BN representing nodes influencing SMM.

The importance of various variables was analyzed using DWI (Eq 2) based on the established BN in Fig 3. The weights in Eq 2 are equal to the product of the strengths of the arcs in the path [54]. Fig 4 shows the heatmap of BN for the target node SMM (shown in red). The BN in Fig 4 excludes arcs that were not plausible based on prior knowledge. As shown in Fig 4, none of the county-level variables have an influence on the probability SMM. Hypertensive disorder of pregnancy has the highest influence on the likelihood of SMM followed by anemia, pre-existing hypertension and prior cesarean. Other less influential variables include diabetes mellitus complicating pregnancy, diabetes mellitus, parity, mother’s race, mother’s age and obesity complication in pregnancy.

### Understanding racial disparities in SMM

In order to understand the racial disparities in SMM, we performed several conditional probability queries, where the conditions are on the distribution of one or more variables, but the probabilistic relationships in the BN are left intact. This type of BN is not considered a causal BN, and interpreting BNs in terms of causality is not necessary for extracting meaningful information learned from the structure [34]. Due to relatively large number of variables in the BN, we used an approximate method called likelihood weighting that uses Monte Carlo simulations to sample from the global distribution to estimate the marginal posterior distribution of the event given evidence (i.e., specific values for a node). We used the “cpquery” function in “bnlearn” to perform the inference required to calculate the conditional probabilities of SMM. We computed the conditional probability of SMM given that a woman was non-Hispanic White, non-Hispanic Black, Hispanic, or of another race. Table 4 shows the odds ratio of SMM across minority groups compared to non-Hispanic White women using BN and a classical logistic regression approach.

**Table 4:** Comparison of SMM risk using BN approximate inference and logistic regression.

Event	Evidence	BN Model		Logistic Regression	
		Odds ratio	CI	Odds ratio	CI
SMM	Non-Hispanic White	1.00 (Ref)	-	1.00 (Ref)	-
	Non-Hispanic Black	1.15	[1.14 1.17]	1.30	1.13⁃1.49
	Hispanic	1.04	[1.02 1.05]	0.96	0.77⁃1.18
	Other	1.06	[1.05 1.08]	1.14	0.88⁃1.47

In both models, Black women have elevated odds ratio for SMM compared to White women. Hispanic and other racial minorities represent slightly higher odds compared to White women based on the BN model compared to the findings from the logistic regression.

Race was not in the Markov blanket of SMM; however, several important comorbid conditions were in the Markov blanket of the race such as anemia, preexisting hypertension, and infectious and parasitic conditions complicating pregnancy. Several other variables including marital status and percent African American population in the county were the children nodes in the Markov blanket of race. Among the comorbid conditions, we inferenced on the two conditions that was found in the Markov blanket of SMM (i.e., anemia and hypertensive disorder of pregnancy). Anemia and preexisting hypertension (a parent node to hypertensive disorder of pregnancy) were also found in the Markov blanket of race. The conditional probability of anemia and hypertensive disorder of pregnancy for non-Hispanic Black women were 0.14 and 0.13, respectively. These probabilities were lower among non-Hispanic White (0.07 and 0.11), Hispanic (0.11 and 0.09), and other races (0.12 and 0.09).

### Understanding disparities in prenatal care

Early onset of prenatal care is linked to increased likelihood of healthy pregnancy through screening and management of risk factors. A few studies have shown an association between fewer prenatal visits and poor infant outcomes such as low birth weight, preterm birth and infant mortality [58,59]. However, the relationship between prenatal visits and maternal outcomes are less established [8]. We found slightly higher odds for SMM among women with insufficient prenatal care use compared to women with sufficient prenatal care (1.01, 95% CI: 1.00–1.02). We further investigated the disparities in receipt of the prenatal care across race and ethnicity. The odds of having insufficient prenatal care visits was significantly higher among non-Hispanic Black women as compared to other races (Table 5).

**Table 5:** Disparities in prenatal care use.

Event	Evidence	Odds Ratio	CI
Insufficient prenatal care visit	Non-Hispanic White (Ref)		1.00 (Ref)
	Non-Hispanic Black	1.23	[1.22 1.24]
	Hispanic	1.04	[1.03 1.05]
	Other	1.03	[1.02 1.04]

## Discussion

This study used a BN approach to evaluate the association between different clinical, maternal demographic, and area-level factors on the probability of SMM. While only 2% of the patient population in our study experienced SMM, BN highlighted important points regarding the associations among demographics, maternal and birth-related variables, comorbid conditions, and SMM. Of significance, we found that comorbid conditions were more likely to be associated with SMM than other maternal and area-level factors. Anemia and hypertensive disorder of pregnancy were revealed as being associated with SMM by virtue of its membership in the Markov blanket. The Markov blanket of a node represents all the variables that can give information about the variable represented by a node [34]. The Markov blanket of a node consists of its parents, children, and its children’s parents (co-parents) [34]. As displayed in Fig 4, anemia, and hypertensive disorder of pregnancy are parents to SMM and can be stratified. In particular these variables block the effects of other variables. If stratified, these variables (anemia and hypertensive disorder of pregnancy) will break the link between all other variables and SMM. We additionally evaluated the importance of the nodes in relation to the target node (SMM) on the basis of network structure using a DWI index, for which hypertensive disorder of pregnancy, anemia, and preexisting hypertension were the three highest impacting factors.

While race was not in the Markov blanket of SMM, we found that anemia and preexisting hypertension were in the Markov blanket of race. Higher conditional probabilities for anemia and hypertensive disorder given race showed that large disparities exist in anemia prevalence among non-Hispanic Black women compared to non-Hispanic White. This differential has been explained among older Black adults [60] as well as younger healthy Blacks compared to Whites [61]. Several other studies explore these racial differences as well as whether correlates of anemia differ between Black and White individuals [62–65], and we add to this literature by quantitively capturing these racial differences among birthing women.

This study had some limitations. First, as previously described, the BN approaches used in this paper were exploratory in nature and do not suggest causality. Second, there are some characteristics that are absent from the data that may ultimately impact maternal health, such as income or access to health foods; however, we utilized variables such as WIC participation and insurance type (i.e., Medicaid vs private) in order to simulate some aspects of socioeconomic status. Finally, this study uses the CDC’s definition of SMM. While this definition is utilized among dozens of studies, we recognize that many important maternal health outcomes are not included in this definition, including near miss death due to suicide and self-harm, accidental drug overdose, interpersonal violence or homicide, and psychological trauma [66,67]. Additional BN analyses should consider whether there are clinical and demographic variables that drive disparities in other maternal health outcomes and morbidities.

## Conclusions

In this study, we used variables from birth certificates linked with medical claims of women giving birth between April 1^st^, 2014 through November 19^th^, 2017. Variables in the study include maternal and birth-related variables, county-level risk factors and comorbid conditions. SMM was identified using the surveillance algorithm provided by the CDC for the day of the delivery through 42 days postpartum. Due to severe imbalance class issue (i.e., only 2% experienced SMM), we first balanced the dataset and then used various structure learning algorithms (score-based, constraint-based and hybrid algorithms) to choose the best learning algorithm. The best network structure was chosen based on the structure with the highest occurrence of an arc between any two nodes that occurred more than 70% of the time in implementation of each algorithm. We then measured the arc strengths using 1000 bootstrapped samples and used DWI index to identify the most influential nodes on the target node. DWI and the Markov blanket of SMM both identified anemia and hypertensive disorder of pregnancy as the most relevant conditions associated with SMM and with disparities in SMM. Our findings suggest the potential for improved care and treatment for women with anemia and hypertensive disorder of pregnancy during the pregnancy and even prior to pregnancy to reduce SMM overall and among women of color.

## Supporting information

S1 Table Pregnancy-related and comorbid conditions and corresponding ICD-9-CM/ICD-10-CM codes. (PDF)
