# Supplementary material for: Understanding racial disparities in severe maternal morbidity using Bayesian network analysis
Source: PLoS One. 2021 Oct 27;16(10):e0259258. doi: 10.1371/journal.pone.0259258 (PMC8550416; doi:10.1371/journal.pone.0259258)
Supplement: S1 Table — (PDF) [file pone.0259258.s001.pdf]

| Condition | ICD-9 | ICD-10 |
|---|---|---|
| Multiple gestation | V27.2-V27.7, 651.x | |
| Insufficient prenatal care | V23.7x | |
| Prior cesarean | 654.2x | |
| Anemia | 648.2 | O9081, O99011, O99013, O99019, O9902, O9903 |
| Asymptomatic bacteriuria in pregnancy | 646.5 | O2300, O2310, O2320, O2330, O2340, O2341, O2342, O2343, O23519, O23529, O23599, O2390, O2391, O2392, O2393, O9089 |
| Bariatric surgery status | 649.2 | O99840, O99841, O99842, O99843, O99844, O99845 |
| Bone and joint disorders | 648.7 | O330 |
| Cervical shortening | 649.7 | O26872, O26873, O26879 |
| Chronic heart disease | 745.0x-747.4x, 648.5x | I050, I051, I052, I058, I060, I061, I062, I068, I069, I071, I072, I078, I080, I080, I088, I089, I091, I0989, I201, I208, I208, I209, I2510, I2510, I252, I253, I2541, I2542, I255, I25810, I25810, I25810, I25810, I25811, I25812, I2582, I2583, I2584, I2589, I259, I259, I270, I271, I272, I2781, I2782, I2789, I279, I340, I348, I350, I351, I352, I358, I359, I360, I368, I370, I378, I38, I38, I39, I421, I422, I423, I424, I425, I427, I428, I428, I43, I43, I440, I441, I441, I442, I4430, I4430, I4439, I444, I445, I4460, I4469, I447, I4510, I452, I452, I452, I453, I454, I455, I456, I456, I4581, I4589, I459, I5022, I5023, I5032, I5033, I5042, I5043 |
| Chronic liver disease | 571.xx, 572.xx | K700, K7010, K7030, K709, K7210, K7290, K7290, K7291, K730, K732, K738, K739, K740, K741, K743, K744, K745, K7460, K7469, K750, K751, K754, K760, K766, K767, K7689, K769 |
| Chronic renal disease | 581.x-583.x, 585.x, 587.x, 588.x, 646.2x | N022, N032, N033, N035, N038, N039, N040, N043, N044, N048, N049, N052, N055, N058, N059, N08, N110, N118, N171, N172, N181, N182, N183, N184, N185, N186, N189, N250, N251, N2581, N2589, N259, N269, N3010, N3011, N3020, N3021 |
| Renal disease complicating pregnancy | 646.2 | O26831, O26832, O26833, O26839, O9089 |
| Chronic respiratory disease | 491.xx-496.xx | J410, J411, J418, J42, J439, J440, J441, J449, J4520, J4521, J4522, J45901, J45902, J45909, J45990, J45991, J45998, J471, J479, J670, J671, J672, J673, J674, J675, J676, J677, J678, J679 |
| Coagulation defects complicating pregnancy | 649.3 | O99111, O99112, O99113, O99119, O9912, O9913 |
| Congenital heart disease | 745.0x-747.4x, 648.5x | Q200, Q201, Q203, Q204, Q205, Q208, Q209, Q210, Q211, Q212, Q213, Q218, Q219, Q220, Q221, Q222, Q223, Q225, Q229, Q230, Q231, Q232, Q233, Q234, Q238, Q240, Q241, Q242, Q243, Q244, Q245, Q246, Q248, Q249, Q251, Q252, Q253, Q254, Q255, Q256, Q2571, Q2572, Q2579, Q260, Q261, Q262, Q263, Q268, Q269 |
| Congenital cardiovascular disorders complicating pregnancy | 648.5 | O99411, O99412, O99412, O99413, O99419, O9942, O9943 |
| Diabetes mellitus complicating pregnancy | 648.0, 648.8 | O24319, O2432, O24419, O24429, O24439, O24911, O24912, O24913, O2492, O2493, O99810, O99814, O99815, O99815 |
| Diabetes mellitus | 249.xx, 250.xx | E0801, E0810, E0811, E0821, E08311, E08319, E0836, E0839, E0840, E0841, E0842, E0843, E0844, E0849, E0851, E08610, E08618, E08620, E08621, E08622, E08628, E08630, E08638, E08641, E0865, E0869, E088, E089, E0901, E0910, E0911, E0921, E09311, E09319, E0936, E0939, E0940, E0941, E0942, E0943, E0944, E0949, E0951, E09610, E09618, E09620, E09621, E09622, E09628, E09630, E09638, E09641, E09649, E0965, E0969, E098, E098, E099, E1010, E1011, E1021, E1029, E10311, E10319, E1036, E1039, E1040, E1051, E10618, E10620, E10621, E10622, E10628, E10630, E10638, E10641, E10649, E1065, E1069, E108, E109, E1100, E1101, E1121, E1129, E11311, E11319, E1136, E1139, E1140, E1151, E11618, E11620, E11621, E11622, E11628, E11630, E11638, E11641, E11649, E1165, E1169, E118, E119, E1300, E1310, E1311, E1339, E1340, E1341, E1342, E1343, E1344, E1349, E1359, E13620, E13621, E13622, E13628, E13638, E13641, E13649, E1365, E1369, E138, E139 |
| Drug dependence complicating pregnancy | 648.3 | O99320, O99321, O99322, O99323, O99324, O99325 |

| | | |
|---|---|---|
| Epilepsy complicating pregnancy | 649.4 | O99350, O99351, O99352, O99353, O99354, O99355 |
| Excessive weight gain in pregnancy | 646.1 | O1200, O1201, O1202, O1203, O1220, O1221, O1222, O1223, O2600, O2601, O2602, O2603 |
| Infections of genitourinary tract in pregnancy | 646.6 | O2300, O2310, O2320, O2330, O2340, O23519, O23529, O23599, O2390, O2391, O2392, O2393, O8611, O8613, O8619, O8620, O8621, O8622, O8629 |
| Habitual aborter currently pregnant | 646.3 | O2620, O2621, O2622, O2623 |
| Hemorrhage in early pregnancy | 640 | O200, O208, O209 |
| Hepatitis C | V02.61, 070.2x, 070.3x | B1710, B1711, B182, B1920, B1921, Z2252 |
| Deep vein thrombosis | V12.51 | Z86718 |
| Hypercoagulable state | 289.81, 289.82 | D6851, D6852, D6859, D6861, D6862, D6869 |
| Hypertensive disorder of pregnancy | 642.3x-642.6x, 642.9x | O131, O132, O133, O139, O1400, O1402, O1403, O1410, O1412, O1413, O1420, O1422, O1423, O1490, O1492, O1493, O1502, O1503, O151, O152, O159, O161, O162, O163, O169 |
| Infectious and parasitic conditions complicating pregnancy | 647 | O98011, O98012, O98013, O98019, O9802, O9803, O98111, O98112, O98113, O98119, O9812, O9813, O98211, O98212, O98213, O98219, O9822, O9823, O98311, O98312, O98313, O98319, O9832, O9833, O9842, O9843, O98511, O98512, O98512, O98513, O98519, O9852, O9853, O98611, O98612, O98613, O98619, O9862, O9863, O98811, O98812, O98813, O98819, O9882, O9883, O98911, O98912, O98913, O98919, O9892, O9893, O99834, O99835 |
| Liver and biliary tract disorders in pregnancy | 646.7 | O26611, O26612, O26613, O26619, O2662 |

| | | |
|---|---|---|
| Malignancy | 140.x-208.x | C000, C001, C002, C003, C004, C005, C006, C008, C01, C020, C021, C022, C023, C024, C028, C029, C030, C031, C039, C040, C041, C048, C049, C050, C051, C052, C059, C060, C061, C062, C0689, C069, C07, C080, C081, C089, C089, C090, C091, C099, C100, C101, C102, C103, C104, C108, C108, C109, C110, C111, C112, C113, C118, C119, C12, C130, C131, C132, C138, C139, C140, C142, C148, C148, C153, C153, C154, C154, C155, C155, C158, C159, C160, C161, C162, C163, C164, C165, C166, C168, C169, C170, C171, C172, C173, C178, C179, C180, C181, C182, C183, C184, C185, C186, C187, C188, C189, C19, C20, C210, C211, C218, C220, C221, C222, C227, C228, C229, C23, C240, C241, C248, C249, C250, C251, C252, C253, C254, C257, C258, C259, C260, C261, C269, C300, C301, C310, C311, C312, C313, C318, C319, C320, C321, C322, C323, C328, C329, C33, C3400, C3410, C342, C3430, C3480, C3490, C37, C380, C381, C382, C383, C384, C388, C390, C399, C4000, C4010, C4020, C4030, C410, C411, C412, C413, C414, C419, C430, C4310, C4320, C4330, C4331, C4339, C434, C4359, C4360, C4370, C438, C439, C4400, C4401, C4402, C4409, C44101, C44111, C44121, C44191, C44201, C44211, C44221, C44291, C44300, C44301, C44309, C44310, C44311, C44319, C44320, C44321, C44329, C44390, C44391, C44399, C4440, C4441, C4442, C4449, C44500, C44501, C44509, C44510, C44511, C44519, C44520, C44521, C44529, C44590, C44591, C44599, C44601, C44611, C44621, C44691, C44701, C44711, C44721, C44791, C4480, C4481, C4482, C4489, C4490, C4491, C4492, C4499, C460, C461, C462, C463, C464, C4650, C467, C469, C478, C480, C481, C482, C488, C490, C4910, C4920, C493, C494, C495, C496, C498, C499, C50019, C50029, C50119, C50219, C50319, C50419, C50519, C50619, C50819, C50919, C50929, C510, C511, C512, C519, C52, C530, C531, C538, C539, C540, C541, C542, C543, C548, C549, C55, C569, C5700, C5710, C5720, C573, C574, C574, C577, C578, C579, C58, C600, C601, C602, C608, C609, C61, C6200, C6210, C6290, C6300, C6310, C632, C637, C638, C639, C649, C659, C669, C670, C671, C672, C673, C674, C675, C676, C677, C678, C679, C680, C681, C688, C689, C6900, C6910, C6920, C6930, C6940, C6950, C6950, C6960, C6980, C6990, C700, C701, C709, C710, C711, C712, C713, C714, C715, C716, C717, C718, C719, C720, C721, C7250, C729, C729, C73, C7490, C750, C751, C752, C753, C754, C755, C758, C759, C760, C761, C762, C763, C7640, C7650, C768, C770, C771, C772, C773, C774, C775, C778, C779, C7800, C781, C782, C7839, C784, C785, C786, C787, C787, C7889, C7900, C7911, C7919, C792, C7931, C7932, C7949, C7951, C7952, C7960, C7970, C7981, C7982, C7989, C800, C801, C802, C8100, C8101, C8102, C8103, C8104, C8105, C8106, C8107, C8108, C8109, C8110, C8111, C8112, C8113, C8114, C8115, C8116, C8117, C8118, C8119, C8120, C8121, C8122, C8123, C8124, C8125, C8126, C8127, C8128, C8129, C8130, C8131, C8132, C8133, C8134, C8135, C8136, C8137, C8138, C8139, C8140, C8141, C8142, C8143, C8144, C8145, C8146, C8147, C8148, C8149, C8170, C8171, C8171, C8171, C8172, C8172, C8172, C8173, C8173, C8173, C8174, C8174, C8174, C8175, C8175, C8175, C8176, C8176, C8176, C8177, C8177, C8177, C8178, C8178, C8178, C8179, C8179, C8179, C8190, C8191, C8192, C8193, C8194, C8195, C8196, C8197, C8198, C8199, C8290, C8291, C8292, C8293, C8294, C8295, C8296, C8297, C8298, C8299, C8310, C8311, C8312, C8313, C8314, C8315, C8316, C8317, C8318, C8319, C8330, C8331, C8331, C8331, C8332, C8332, C8332, C8333, C8333, C8333, C8334, C8334, C8334, C8335, C8335, C8335, C8336, C8336, C8336, C8337, C8337, C8337, C8338, C8338, C8338, C8339, C8339, C8339, C8350, C8351, C8352, C8353, C8354, C8355, C8356, C8357, C8358, C8359, C8370, C8371, C8372, C8373, C8374, C8375, C8376, C8377, C8378, C8379, C8380, C8380, C8380, C8381, C8381, C8381, C8382, C8382, C8382, C8383, C8383, C8383, C8384, C8384, C8384, C8385, C8385, C8385, C8386, C8386, C8386, C8387, C8387, C8387, C8388, C8388, C8388, C8389, C8389, C8389, C8400, C8401, C8402, C8403, C8404, C8405, C8406, C8407, C8408, C8409, C8410, C8411, C8412, C8413, C8414, C8415, C8416, C8417, C8418, C8419, C8440, C8441, C8442, C8443, C8444, C8445, C8446, C8447, C8448, C8449, C8460, C8461, C8462, C8463, C8464, C8465, C8466, C8467, C8468, C8469, C8470, C8471, C8472, C8473, C8474, C8475, C8476, C8477, C8478, C8479, C8493, C8580, C8581, C8582, C8583, C8584, C8585, C8586, C8587, C8588, C8589, C888, C888, C9000, C9001, C9002, C9010, C9011, C9012, C9020, C9021, C9022, C9030, C9031, C9032, C9100, C9101, C9102, C9110, C9111, C9112, C9140, C9140, C9140, C9140, C9140, C9140, C9140, C9140, C9140, C9141, C9190, C9191, C9192, C91Z0, C91Z0, C91Z1, C91Z1, C91Z2, C91Z2, C9200, C9201, C9202, C9210, C9211, C9212, C9220, C9221, C9222, C9230, C9231, C9232, C9240, C9241, C9242, C9250, C9251, C9252, C9290, C9291, C9292, C92Z0, C92Z1, C92Z2, C9300, C9301, C9302, C9310, C9311, C9312, C9390, C9390, C9391, C9391, C9392, C9392, C93Z0, C93Z1, C93Z2, C9400, C9401, C9402, C9420, C9421, C9422, C9430, C9431, C9432, C9480, C9481, C9482, C9500, C9501, C9502, C9510, C9511, C9512, C9590, C9590, C9590, C9591, C9592, C960, C962, C964, C969, C969, C96A, C96Z, D030, D0310, D0311, D0312, D0320, D0321, D0322, D0330, D0339, D034, D0351, D0352, D0359, D0360, D0361, D0362, D0370, D0371, D0372, D038, D039, D45 |

| | | |
|---|---|---|
| Mental health disorders | 293.81-293.84, 295.00-295.05, 295.10-295.15, 295.20-295.25, 295.30-295.35, 295.40-295.45, 295.50-295.55, 295.60-295.65, 295.70-295.75, 295.80-295.85, 295.90-295.95, 296.00-296.06, 296.10-296.16, 296.20-296.26, 296.30-296.36, 296.40-296.46, 296.50-296.56, 296.60-296.66, 296.7x, 296.80, 296.81, 296.82, 296.89, 296.90, 296.99, 297.0x-297.3x, 297.8x-298.4x, 298.8x, 298.9x, 300.00-300.02, 300.09, 300.10, 300.20-300.23, 300.29, 300.3x-300.5x, 300.89, 300.9x, 301.0x, 301.10-301.13, 301.20-301.22, 301.3x, 301.4x, 301.50, 301.51, 301.59, | F060, F062, F0630, F064, F17200, F200, F201, F202, F205, F2081, F2081, F2089, F209, F21, F22, F23, F24, F259, F28, F29, F3010, F3011, F3012, F3013, F302, F303, F304, F308, F3110, F3111, F3112, F3113, F312, F3130, F3131, F3132, F314, F315, F3160, F3161, F3162, F3163, F3164, F3173, F3174, F3175, F3176, F3177, F3178, F3181, F319, F320, F321, F322, F323, F324, F325, F328, F329, F330, F331, F332, F333, F3341, F3342, F339, F340, F341, F348, F39, F4001, F4002, F4010, F40218, F40240, F40241, F408, F409, F410, F411, F418, F419, F42, F430, F4310, F4312, F4489, F449, F458, F488, F489, F600, F601, F602, F603, F604, F605, F606, F607, F6081, F6089, F609, F938, F938, F938, F938, F99, R457 |

| | | |
|---|---|---|
| | 301.6x, 301.7x, 301.81-301.84, 301.89, 301.9x, 308.0x-308.4x, 308.9x, 309.81, 311.xx, 313.0x, 313.1x, 313.21, 313.22, 313.3x, 313.82, 313.83 | |
| Mental disorders complicating pregnancy | 648.4 | O906, O99340, O99341, O99342, O99343, O99344, O99345, O99345 |
| Obesity | 278.0x | E6601, E662, E663, E669 |
| Pregnancy-related Obesity | 649.1x | O99210, O99211, O99212, O99213, O99214, O99215 |
| Other cardiovascular diseases complicating pregnancy | 648.6 | O99411, O99412, O99413, O99419, O9942, O9943 |
| Peripheral neuritis in pregnancy | 646.4 | O26821, O26822, O26823, O26829, O9089 |
| Placenta previa | 641.0x, 641.1x | O4400, O4401, O4402, O4403, O4410, O4411, O4412, O4413 |
| Preexisting hypertension | 401.x-405.x, 642.0x-642.2x, 642.7x | I10, I119, I120, I129, I130, I1310, I1311, I132, I150, I158, O10011, O10012, O10013, O10019, O1002, O1003, O10111, O10112, O10113, O10119, O1012, O1013, O10211, O10212, O10213, O10219, O1022, O1023, O10311, O10312, O10313, O10319, O1032, O1033, O10411, O10412, O10413, O10419, O1042, O1043, O10911, O10912, O10913, O10919, O1092, O1093, O111, O112, O113, O119, O152 |
| Sickle cell disease | 282.4x, 282.6x | D560, D561, D562, D563, D565, D568, D569, D5700, D571, D5720, D57219, D5740, D57419, D5780, D57819 |
| Spine abnormalities | 737.3x-737.4x, 741.x | M4010, M4050, M4100, M4120, M4130, M4140, M4150, M4180, M419, M438X9, M965, Q050, Q051, Q052, Q054, Q055, Q056, Q057, Q058, Q0701, Q0702, Q0703 |
| Substance use Alcohol | 303.xx, 305.0x, 291.xx, 357.5, 790.3, 425.5, 535.3, 980.0, 790.3, 760.71, E86.00, E86.08, E86.09 | |
| Substance use Cannabis | 304.3x, 305.2x | |
| Substance use Cocaine | 304.2x, 305.6x, 760.75, 970.81 | |
| Substance use Opiates | 304.0x, 304.7x, 305.5x, 965.00-965.02, 965.09, E85.00 | |

| | | |
|---|---|---|
| Substance use Other | 304.1x, 304.4x, 304.5x, 304.6x, 305.3x, 305.4x, 305.7x, 305.8x, 969.6x, 760.73, E85.41 | |
| Substance use Unspecified | 304.8x, 304.9x, 305.9x, 648.3x | |
| Systemic lupus erythematosus | 710.0x | |
| Tobacco use disorder complicating pregnancy | 649.0 | |
| Multiple gestation | V27.2-V27.7, 651.x | |
| Insufficient prenatal care | V23.7x | |
| Prior cesarean | 654.2x | |
